# Severe electrolytes disorders with the interstitial kidney alterations in the patient with the history of total thyroidectomy and parathyroidectomy: possible role of vitamin D deficiency

**DOI:** 10.1002/ccr3.1500

**Published:** 2018-04-06

**Authors:** Emi Kawakita, Keizo Kanasaki, Taro Hirai, Shin‐ichi Tsuda, Ai Watanabe, Kyoko Nitta, Munehiro Kitada, Yoshio Ogura, Yuta Takagaki, Mizue Fujii, Takako Nagai, Keiji Shimada, Susumu Takagi, Yuiko Mizunuma, Itaru Monno, Fujimoto Shino, Hiroshi Minato, Nobuhiko Miyatake, Atsushi Nakagawa, Daisuke Koya

**Affiliations:** ^1^ Department of Diabetology & Endocrinology Kanazawa Medical University Uchinada Ishikawa 920‐0293 Japan; ^2^ Division of Anticipatory Molecular Food Science and Technology Medical Research Institute Kanazawa Medical University Uchinada Ishikawa 920‐0293 Japan; ^3^ Department of Hematology and Immunology Kanazawa Medical University Uchinada Ishikawa 920‐0293 Japan; ^4^ Department of Pathology and Laboratory Medicine Kanazawa Medical University Uchinada Ishikawa 920‐0293 Japan; ^5^ Department of Nephrology Kanazawa Medical University Uchinada Ishikawa 920‐0293 Japan

**Keywords:** Hypokalemia, Mg, ROMK, vitamin D

## Abstract

Vitamin D plays vital role for the health, and its deficiency has been implicated in the diverse pathological conditions such as hypomagnesemia and abnormal immune system. Here, we present a case of severe electrolytes disorders (hypokalemia and hypomagnesemia etc.) and kidney damages associated with vitamin D deficiency.

## Introduction

Although vitamin D is essential for health and plays vital roles in electrolyte homeostasis, vitamin D deficiency is a relatively common nutrition disorder 1. In the United Kingdom, 35.7% of Black, 59.6% of Asian, and 19.6% of White display 25(OH)D3 levels lower than 30 nmol/L 2. Similar trend has been reported in worldwide 3. In general, vitamin D is recognized as vital molecule in the homeostasis of calcium and phosphate levels; vitamin D deficiency has been implicated in the diverse pathological conditions such as hypomagnesemia 4, auto‐antibodies induction 5, and *Helicobacter pylori* infection 6.

Electrolyte abnormalities, such as hypokalemia and hypomagnesemia, are often observed in the clinic. Indeed, magnesium exhibits critical roles in the normal homeostasis of potassium via suppression of the renal outer medullary potassium channel (ROMK)‐mediated potassium secretion into the renal tubule 7. We experienced a case of severe electrolyte disorders, including hypokalemia, hypomagnesemia, and hypocalcemia, associated with vitamin D deficiency.

## Case History

A 70‐year‐old woman with a history of thyroidectomy was transferred to the emergency unit of our hospital due to muscle weakness and tetany.

More than 30 years ago, the patient underwent a thyroidectomy but lacked an apparent forearm operation scar that suggested parathyroid autotransplantation. She was prescribed sodium levothyroxine 100 μg and 1,25‐dihydroxyvitamin D3 2 μg per day, although occasionally she took prescribed drugs only when she felt fatigues. The dose of sodium levothyroxine remained unchanged for more than 20 years even though regular testing of thyroid function indicated relative hypothyroidism (free T3: 1.96 pg/mL; reference range 2.3–4.0, free T4: 1.02 ng/dL; 0.9–1.7, and TSH: 59.581 *μ*IU/mL; 0.50–5.00). Her uterus had been dissected due to myoma uteri. She had a 5‐year history of bipolar disorder and was treated with aripiprazole. She had been prescribed sodium valproate until approximately 2 weeks prior to admission to this hospital; the final monitoring for the valproate concentration was performed approximately 6 months ago, and the total valproate level was 77 *μ*g/mL (the therapeutic range; 50–100 *μ*g/mL). She also took losartan potassium (50 mg/day) and nifedipine (40 mg/day) for hypertension and pravastatin sodium (5 mg/day) for dyslipidemia. She did not take any supplements or ingest possible glycyrrhizin or licorice‐containing food. She is a housewife and sleeps well. Her appetite is normal; she often eats premade side dishes from the supermarket. She does not exercise regularly. Over the past 5 years, she tended to feel fatigued due to malaise. She also had diabetes mellitus without a known duration (HbA1c: 6.6% with random glucose measurements over 200 mg/dL).

Her father died by committing suicide, and her mother passed away when she was 3 years old, although the details were not known. She has a younger brother and sister, neither of which have particular diseases. Her two daughters and son were all free from any diseases.

On the day of admission, she experienced muscle weakness in both lower limbs during her afternoon shopping and felt an absolute loss of muscle power when she returned home. Subsequently, she experienced loss of grip power and muscle twitching. These symptoms progressively worsened, and her family requested an emergency assistance and an ambulance. She experienced similar but mild symptoms 1–2 times per week; typically, the symptoms were relieved without any specific intervention, although occasionally she took prescribed drugs. When arriving at the emergency unit of our hospital, she was alert, her blood pressure was 116/76 mmHg, her heart rate was 92/min regularly, and her respiratory rate was 18/min. She had a normal body temperature of 36.5°C. Her height was 154 cm, and her weight was 75 kg (body mass index: 31.6 kg/m^2^). Her grip power was 5 (rt) and 0 (lt) kg. Her muscular power assessed using manual muscle test (MMT) resulted in 3 points in both limbs. Both hands appeared to be clenched fists. Nonpitting edema was noted in the bilateral dorsum of the hand, and a pretibial lesion.

Arterial blood gas (ABG) analysis revealed alkalemia with metabolic alkalosis (pH 7.537, HCO_3_: 34.4 mmol/L) without respiratory failure (Table 1). She had hypokalemia, hypochloremia, and hypocalcemia (Table 1). The ECG analysis demonstrated a decrease in the amplitude of the T wave and an increase in the amplitude of the U wave in leads V4 to V6. Hypomagnesemia was also diagnosed the morning after her admission (Table 1). The urine electrolytes analysis revealed an inappropriate urine loss of potassium (transtubular potassium gradient; TTKG: 4.73). Additionally, urine loss of chloride and magnesium was inappropriately induced (fractional excretion of chloride; FECl: 2.07% and fractional excretion of magnesium; FEMg; 16.2% at the next day after admission), whereas urine calcium loss was not obvious at admission (urine calcium/creatinine ratio: 0.09 mg/mg at admission and 0.26 mg/mg on the next day). Hypophosphatemia was also present. She had hypothyroidism, probably due to her poor adherence. Her parathyroid hormone level were below the detection limit. Lactic acid was high on the first ABG analysis, but this observation immediately disappeared, suggesting that it was the result of a muscle cramp. Her complete blood cell count and coagulation tests were normal. The urine microscopy analysis revealed no apparent urinary tract infection. (urine sediment: white cells 1–4 counts/high power fields (HPF);reference range, less than 5 counts/HPF, red cells 1–4 counts/HPF; reference range, less than 5 counts/HPF, and bacterial reaction was negative.)

**Table 1:** Laboratory date during admission

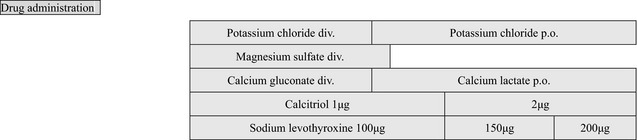										
Variable	Reference range	Day 1^a^	Day 2	Day 3	Day 5	Day 9	Day 12	Day 18	Day 22	Day 31
Arterial blood gas analysis while the patient was breathing ambient air (Day 2: Venous blood gas data)										
pH	7.35–7.45	7.537	7.493			7.445				
PaCO_2_ (mmHg)	35–45	40.7	52.7			39.2				
PaO_2_ (mmHg)	83–108	80.4	40.9			71.6				
Base excess (mmol/L)	−2 to +2	10.8	14.7			2.8				
HCO_3_^−^ (mmol/L)	22–26	34.4	40			26.5				
Anion gap (mmol/L)	10–20	14.7	11			14.7				
Na+ (mmol/L)	136–146	143	142			140				
K+ (mmol/L)	3.5–5.0	1.9	2.0			3.9				
Cl− (mmol/L)	98–106	89	93			103				
Glucose (mg/dL)	70–105	158	133			131				
Lactic acid (mg/dL)	4.5–14.4	65	12			16				
Blood/urine										
Na (mEq/L)	138–146	141	144	142	142	141	140	140	140	142
K (mEq/L)	3.6–4.9	1.9	1.9	2.2	2.5	3.2	3.7	4.3	3.8	3.2
Cl (mEq/L)	99–109	90	93	95	95	100	101	100	101	103
Ca^b^ (mg/dL)	8.7–10.3	6.8	7.5	7.3	8.1	8.3	8.7	8.4	8.7	8.3
P (mg/dL)	2.5–4.7		1.9	3.0	3.4	4.1	3.8	5.2	4.6	4.6
Mg (mg/dL)	1.8–2.4		1.5	1.8	1.9	1.8	1.7	2.1	1.9	1.9
BUN (mg/dL)	8–22	11	9	6	6	11	9	15	19	15
Cr (mg/dL)	0.4–0.8	1.13	0.92	0.88	0.87	1.00	0.88	0.97	0.93	0.78
eGFR		37.0	46.4	48.7	49.3	42.3	48.7	43.8	45.8	55.6
FENa (%)	1–2	1.47	2.20	2.46	1.89	0.89	1.53	0.61	0.47	0.63
FEK (%)	<9%^c^	8.88	20.17	15.98	11.35	9.65	4.70	9.56	10.03	9.20
U‐K/U‐Cr (mEq/gCr)	<15^d^	21.7	37.9	32.6	32.6	30.9	43.6	42.4	38.1	41.3
TTKG	<2^e^	4.73	6.23	5.04	4.58	5.31	10.38	6.13	5.95	4.95
FECl (%)	<0.5^f^	2.07	3.42	3.39	2.06	1.12	1.88	0.78	0.64	0.84
U‐Ca/U‐Cr (mg/mg)	0.05–0.20	0.09	0.26	0.33	0.36	0.31	0.36	0.17	0.24	0.32
FEMg (%)	2–3		16.2	15.7	18.6	9.37	4.36	3.34	4.06	6.26
U‐*β*2‐MG (ng/mL)	≦150		12,765			8088		648	36	718
U‐NAG (IU/L)	0.7–11.2		6.2			14.8			12.2	8.3
L‐FABP (*μ*g/gCr)	≦8.4		94.7			68.8			15.4	6

FENa, fractional excretion of sodium; FEK, fractional excretion of potassium; U‐K/U‐Cr, urine potassium concentration and urine creatinine ratio; TTKG, transtubular potassium gradient; FECl, fractional excretion of chloride; U‐Ca/U‐Cr, urine carusium concentration and urine creatinine ratio; FEMg, fractional excretion of magnesium; U‐*β*2‐MG, urine *β*2‐microglobrin; U‐NAG, urine *N*‐acetyl‐*β*‐d‐glucosaminidase; L‐FABP, liver‐type fatty acid‐binding protein.

a Before treatment.

b Correction calcium.

c FEK: should be less than 9% in hypokalemia.

d U‐K/U‐Cr: should be less than 15 in hypokalemia.

e TTKG: reference range, <2 in hypokalemia, >7–10 in hyperkalemia.

f FECl: should be less than 0.5% in hypochloremia.

Our first impression of her electrolyte disorders was Gitelman syndrome (GS)‐like; later, GS was diagnosed by exclusion, as the laboratory test revealed she did not have hyperreninemia with hyperaldosteronism (plasma renin activity 0.2 ng/mL/h, plasma aldosterone 23.9 pg/mL). She also exhibited significant elevation of kidney tubular damage markers, including urine *β*2‐microglobrin, liver‐type fatty acid‐binding protein (L‐FABP), and *N*‐acetyl‐*β*‐d‐glucosaminidase (NAG), several days later. The antinuclear antibody level was as high as ×1280 (discrete‐speckled pattern; reference range, ≦15). The anticentromere antibody level was higher than 240 unit/ml (reference range, ≦10), and the anti‐SSA and SSB antibody tests were negative. The anti‐Sm antibody and anti‐DNA antibody were also negative.

The proteinase 3 (PR3)‐antineutrophil cytoplasmic antibody (ANCA) level was 20.1 unit/mL (reference range, <3.5), and the myeloperoxidase‐ANCA test was negative. The complement values were not suppressed; the C3C was 111 mg/dL (reference range, 86–160), and the C4 was 28 mg/dL (reference range, 17–45). The presence of these auto‐antibodies may suggest atypical Sjögren syndrome, although she did not have either the dry eye or dry mouth symptom. The limited cutaneous form of systemic sclerosis also required a differential diagnosis; however, she did not have calcinosis, Raynaud's phenomenon, esophageal dysmotility, sclerodactyly, telangiectasia, and pulmonary hypertension. She did not have any signs of systemic lupus erythematosus, such as light hypersensitivity, skin rash, and serositis.

After admission, potassium chloride, calcium gluconate hydrate, and magnesium sulfate were administered intravenously for 5 days. Thyroid hormone (sodium levothyroxine, 100 μg per day) and active vitamin D3 (calcitriol, 1 μg per day) were also prescribed. Subsequently, her symptoms were immediately resolved. Her electrolytes abnormalities were also resolved at the 12th day of hospitalization. However, although her electrolytes abnormalities were ameliorated, the presence of abnormally high kidney tubule damage markers in the urine remained. Therefore, a needle biopsy of the kidney was performed on the 22nd day of hospitalization to evaluate histological alterations.

## Renal Biopsy

**Figure 1:**
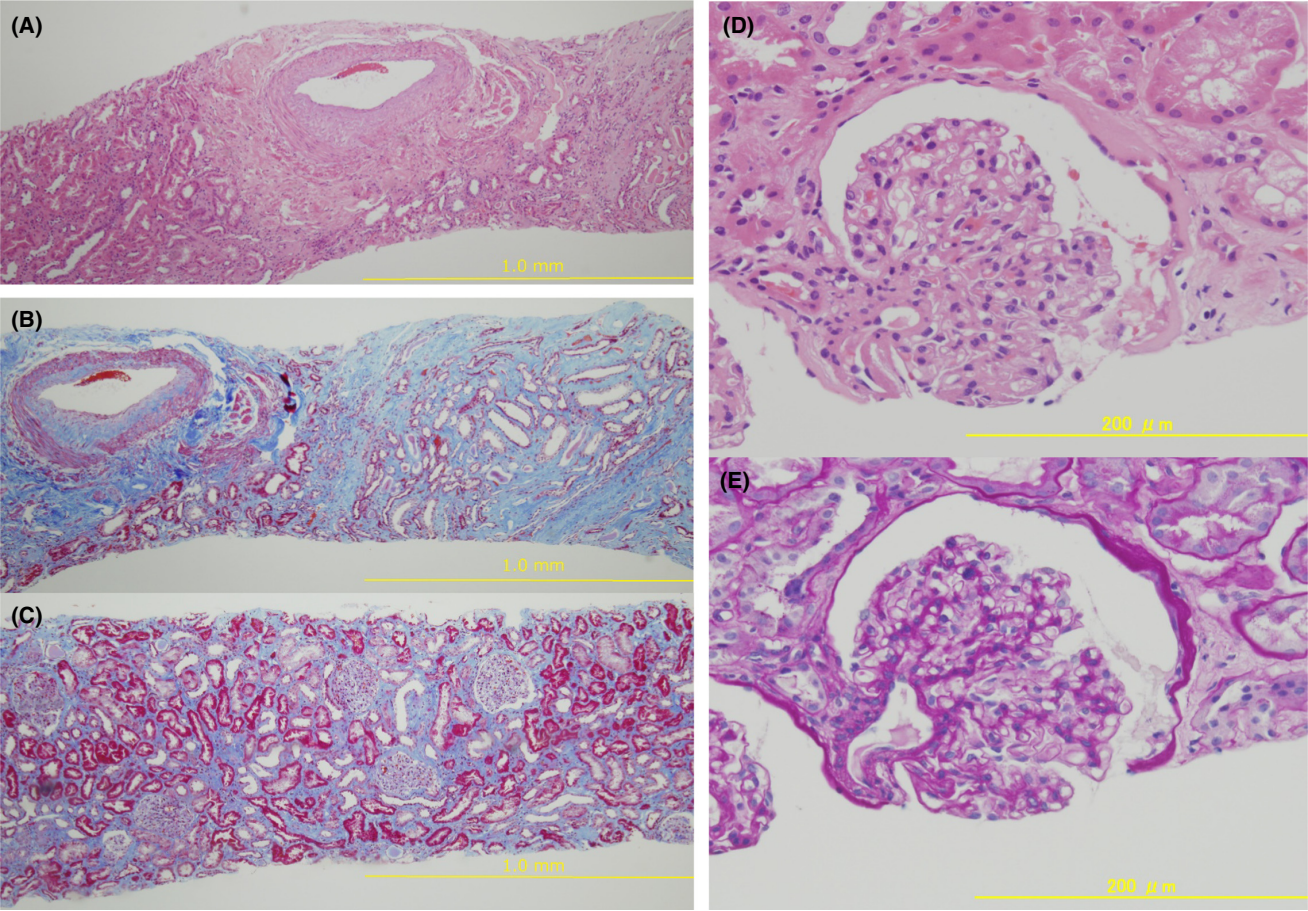
Renal histology. (A–C) Low magnification; (D, E) High magnification. A, D: Hematoxylin and eosin staining, B, C: Masson's trichrome staining. E: Periodic acid–Schiff staining.

We obtained two kidney sections that contained 14 glomeruli, among which three were globally sclerosed. The glomerular structure appeared to be normal in general. We observed no apparent crescent formations, mesangial cell proliferation, and inflammatory cell infiltrate. Some of the glomeruli were collapsed, suggesting possible ischemia. Additionally, some of the glomeruli exhibited polar vasculopathies, suggesting early manifestations of diabetic kidney disease (Fig. 1). The tubular interstitial lesions exhibited severe loss/regeneration of tubular epithelial cells with edema in the tubulointerstitial space. The tubulointerstitial fibrosis was also remarkable. However, no apparent inflammatory cell infiltrate was observed in the interstitial area, and no vasculitis lesions or proliferative, obliterative arteriolar vasculopathy was found.

## Clinical Management

At 36 days after admission, the patient was discharged from this hospital and prescribed potassium chloride (1200 mg), magnesium oxidant (500 mg), calcitriol (1 *μ*g), and sodium levothyroxine (100 *μ*g). At 7 days after discharge, she visited the outpatient clinic of this hospital. Her laboratory findings are shown in Table 2. She still had mild hypokalemia, hypomagnesemia, and hypocalcemia. As the magnesium level had declined after discontinuation of intravenous magnesium for 7 days after admission, her doses of magnesium oxide were increased to 750 mg. Because mild hypertension (137/87 mmHg) was noted, she was prescribed perindopril 2 mg. At the third visit after discharge (9 weeks after discharge), her blood Mg level appeared to be elevated, but mild hypokalemia was still present. Calcium lactate was also initiated at 1 g. At the fourth visit (13 weeks after discharge), she was prescribed 1.5 *μ*g of calcitriol, and subsequently, the dose of calcitriol was increased to 2 μg. At the same time, the amount of calcium lactate was reduced to 0.5 g to reduce calcium excretion in the urine. Currently, the patient is well. She has not felt muscle weakness since the initiation of treatment, and the nonpitting edemas have disappeared from both her hands and legs. During her regular follow‐up, she underwent gastric endoscopy to investigate her esophageal lesion accompanying scleroderma, and mild gastritis with a *H. pylori* infection was found; she was treated with the administration of amoxicillin, clarithromycin, and vonoprazan fumarate. Currently, she does not require any drugs for her depressant or manic states, suggesting that her psychiatric symptoms may have been due to hypothyroidism or electrolytes disorders.

**Table 2:** Laboratory date after discharge

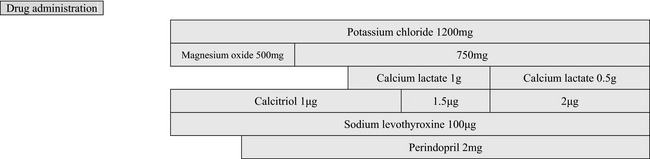								
Variable (blood/urine)	Reference range	1 week	5 weeks	9 weeks	13 weeks	17 weeks	22 weeks	27 weeks
Na (mEq/L)	138–146	142	144	145	142	144	141	143
K (mEq/L)	3.6–4.9	3.4	3.4	3.5	3.7	3.9	3.9	3.9
Cl (mEq/L)	99–109	101	104	103	100	104	103	102
Ca^a^ (mg/dL)	8.7–10.3	6.8	7.5	7.3	8.1	8.3	8.7	8.4
P (mg/dL)	2.5–4.7	3.4	3.8	3.7	4.7	5.3	4.9	4.6
Mg (mg/dL)	1.8–2.4	1.7	1.8	2.1	2.1	2.2	2.2	2.0
Cr (mg/dL)	0.4–0.8	0.79	0.81	0.78	0.77	0.83	0.94	0.91
FEK (%)	<9%^b^	10.5	10.2	7.28	9.53	15.3	19.6	13.9
U‐K/U‐Cr (mEq/gCr)	<15^c^	45.3	42.8	32.7	45.8	72.1	81.4	59.4
TTKG	<2^d^	5.60	6.64	6.67	4.42	10.4	8.8	8.65
FECl (%)	<0.5^e^	0.10	0.10	0.07	1.93	0.11	1.44	1.09
FEMg (%)	2–3	4.87	2.08	7.18	7.06	1.47	8.46	3.57
U‐*β*2‐MG (ng/mL)	≦150	216	450	1361	3066	1782	725	2474
U‐NAG (IU/L)	0.7–11.2	7.3	7.1	15.3	10.1	11.4	7.0	22.2
L‐FABP (*μ*g/gCr)	≦8.4	9.11	7.66	5.65	22.2	4.49	2.14	3.42

a Correction calcium.

b FEK: should be less than 9% in hypokalemia.

c U‐K/U‐Cr: should be less than 15 in hypokalemia.

d TTKG: reference range, <2 in hypokalemia, >7–10 in hyperkalemia.

e FECl: should be less than 0.5% in hypochloremia.

## Discussion

The exact pathophysiological diagnosis of this patient's severe electrolytes disorders was unclear, although her magnesium deficiency was most likely associated with a long history of poor adherence to calcitriol. Magnesium homeostasis associated with vitamin D3 is not completely understood at present. Pointillart et al. 4 reported that vitamin D increased the magnesium level via absorption from the gastrointestinal tract but not reabsorption from the urine. Vitamin D supplementation with magnesium rescued all symptoms, which supported the pathological role of magnesium deficiency in this patient. Magnesium functions as a gatekeeper for potassium excretion through the ROMK channel in the cortical collecting duct (CCD) of the kidney 7. During magnesium deficiency, either high aldosterone or a high sodium chloride level in the urine induces potassium excretion without any defense 7. Correspondingly, on the first day, the patient visited the clinic after discharge, her potassium level was lower at 3.4 mEq/L, and her magnesium was 1.7 mg/dL with TTKG: 5.6 (inappropriate loss of potassium into urine); after increasing the calcitriol dose to 2 μg with magnesium oxide, her potassium level was at 3.9 mEq/L, and her magnesium was 2.2 mg/dl with TTKG: 8.8 (normal excretion of potassium gradients). Some reports indicated that TTKG was not accurate enough maker for potassium homeostasis 8; TTKG was still useful to monitor potassium balance and care for this patient's clinical course. We also utilized U‐K/U‐Cre ratio or FEK, the analyze utilizing serum and urine creatinine levels, for the interpretation of potassium balance in this case and indeed, interpretation based on each analysis was not that different when evaluated by TTKG. However, when evaluating this case, we have been struggling in the best analysis choice for the interpretation of her potassium balance. She had severe interstitial damage with recovery in later time periods; urine creatinine secretion efficacy could be fluctuated due to her histological damage magnitude by which urine creatinine level were theoretically altered. Indeed, approximately 15% of excreted urine creatinine has shown to be derived from secretion by proximal tubular cells 9, 10. Regard with this, there is no clinical assessment possible either to considerate or to differentiate proximal tubule‐derived creatinine in each individual or kidney damaged/recovery phases.

The renal biopsy analysis revealed that this patient exhibited severe tubulointerstitial alterations. Based on the detection of urine markers for tubulointerstitial injuries and renal manifestation by kidney biopsy, the most severe alterations were found on the day of admission, suggesting that the electrolytes disorders, especially hypokalemia, might have influenced histological alterations of the kidney. The lack of signs of pathological lesions suggested an autoimmune disease or ANCA vasculitis. Moreover, a tubulointerstitial injury may increase the delivery of sodium chloride into the CCD. Therefore, tubular damage could have enhanced the electrolytes disorders found in this patient. After discharge during outpatient clinic care interval, urine biomarkers of kidney damage were mildly elevated; we thought this could be the consequence of worsening of diabetes or mild urinary tract infection (bacteriuria with white blood cells in urine sediment: 10–19/high power field).

The contribution of auto‐antibodies in this patient was unclear. Currently, she does not have any apparent characteristics suggestive of collagen diseases, including kidney manifestations. However, her anticentromere antibody level remains higher than 240 unit/mL, and her PR3‐ANCA level has shown a continuously elevated trend (31.3 U/mL at the 2nd visit after discharge). She does not have any signs of hematuria or purpura. Vitamin D deficiency was shown to be associated with increased autoimmune responses 5. Moreover, the induction of auto‐antibodies found in this patient could be associated with the *H. pylori* infection 11, and vitamin D deficiency could potentiate the host to *H. pylori* infection 6. Careful follow‐up is continuously required.

In conclusion, we successfully treated a patient with severe electrolytes disorders characterized by hypokalemia, hypocalcemia, and hypomagnesemia that were possibly associated with vitamin D deficiency due to poor adherence in the patient, who had a past history of thyroidectomy with parathyroidectomy.

## Authorship

EK: involved in patient care, made figure and tables, and participated in writing the manuscript. KK: involved in clinical advice and interpretation of the data and wrote the manuscript. TH, ST, AW, KN, YO, YT, KS, ST, YM, and IM: involved in patient care and discussion. MK, MF, and TN: involved in discussion alone. FS: involved in interpretation of auto‐antibodies. HM and NM: involved in interpretation of renal biopsy. AN and DK: made discussion and intellectual input.

## Conflict of Interest

Authors declare that there are no conflict of interests regarding with this manuscript.
